# What makes community health worker models for tuberculosis active case finding work? A cross-sectional study of TB REACH projects to identify success factors for increasing case notifications

**DOI:** 10.1186/s12960-022-00708-1

**Published:** 2022-03-12

**Authors:** Thu A. Dam, Rachel J. Forse, Phuong M. T. Tran, Luan N. Q. Vo, Andrew J. Codlin, Lan P. Nguyen, Jacob Creswell

**Affiliations:** 1 Friends for International TB Relief, 1/21 Le Van Luong St., Nhan Chinh Ward, Thanh Xuan District, Hanoi, Vietnam; 2 Department of Global Public Health, WHO Collaboration Centre on Tuberculosis and Social Medicine, Karolinska Institutet, Solnavägen 1, 171 77, Solna, Sweden; 3 IRD VN, 68B Nguyen Van Troi, Ward 8, Phu Nhuan District, Ho Chi Minh City, Vietnam; 4 Centre for Development of Community Health Initiatives, 1/21 Le Van Luong St., Nhan Chin Ward, Thanh Xuan District, Hanoi, Vietnam; 5 Stop TB Partnership, Chemin du Pommier 40, 1218 Le Grand-Saconnex, Geneva, Switzerland

**Keywords:** Tuberculosis, Community healthcare workers, TB REACH, Impact evaluation, Active case finding

## Abstract

**Background:**

In the field of tuberculosis (TB), Community Healthcare Workers (CHWs) have been engaged for advocacy, case detection, and patient support in a wide range of settings. Estimates predict large-scale shortfalls of healthcare workers in low- and middle-income settings by 2030 and strategies are needed to optimize the health workforce to achieve universal availability and accessibility of healthcare. In 2018, the World Health Organization (WHO) published guidelines on best practices for CHW engagement, and identified remaining knowledge gaps. Stop TB Partnership’s TB REACH initiative has supported interventions using CHWs to deliver TB care in over 30 countries, and utilized the same primary indicator to measure project impact at the population-level for all TB active case finding projects, which makes the results comparable across multiple settings. This study compiled 10 years of implementation data from the initiative’s grantee network to begin to address key knowledge gaps in CHW networks.

**Methods:**

We conducted a cross-sectional study analyzing the TB REACH data repository (*n* = 123) and primary survey responses (*n* = 50) of project implementers. We designed a survey based on WHO guidelines to understand projects’ practices on CHW recruitment, training, activities, supervision, compensation, and sustainability. We segmented projects by TB notification impact and fitted linear random-effect regression models to identify practices associated with higher changes in notifications.

**Results:**

Most projects employed CHWs for advocacy alongside case finding and holding activities. Model characteristics associated with higher project impact included incorporating e-learning in training and having the prospect of CHWs continuing their responsibilities at the close of a project. Factors that trended towards being associated with higher impact were community-based training, differentiated contracts, and non-monetary incentives.

**Conclusion:**

In line with WHO guidelines, our findings emphasize that successful implementation approaches provide CHWs with comprehensive training, continuous supervision, fair compensation, and are integrated within the existing primary healthcare system. However, we encountered a great degree of heterogeneity in CHW engagement models, resulting in few practices clearly associated with higher notifications.

**Supplementary Information:**

The online version contains supplementary material available at 10.1186/s12960-022-00708-1.

## Background

Since the adoption of the Alma Ata Declaration in 1978 and most recently the Astana Declaration in 2018, primary care with community health workers (CHWs) has been considered a critical path to attain healthcare for all [1, 2]. The specific definition of CHWs can differ, with the term encompassing a diverse group of “lay and educated, formal and informal, paid and unpaid health workers”. These CHWs provide services such as health education and can refer or support individuals and families seeking preventative or curative care. In recognition of the importance of community networks in health, the Global Strategy on Human Resources for Health: Workforce 2030 in 2016 was adopted by the 69th World Health Assembly and subsequently the World Health Organization (WHO) issued guidelines on health policy and system support to optimize CHW programs [3, 4]. These guidelines specifically offered a set of recommendations on CHWs selection, training, management and supervision, career advancement, community embeddedness, and health system support.

Despite substantial evidence on CHWs and their positive impact on communities [5, 6], there remain key knowledge gaps. In particular, the WHO guidelines cited knowledge gaps across six key themes: (1) intervention activities; (2) recruitment/selection; (3) training; (4) compensation/remuneration; (5) supervision; and (6) sustainable integration into the health system [4]. Moreover, studies have decried the lack of evidence on how to integrate and support CHWs within health systems and the “rights and needs of CHWs” [5]. Another review posited that efficacy assessments of CHW projects were carried out under ideal circumstances, leading to a need for more evidence under “real world” conditions [7].

Prior to the COVID-19 pandemic, tuberculosis (TB) was registered as the deadliest infectious disease caused by a single pathogen, resulting in 10 million cases and 1.4 million deaths in 2019 [8]. Meanwhile, the United Nations has made the elimination of TB one of its Sustainable Development Goals [9] and the WHO End TB Strategy aims for a 90% reduction in TB incidence rate by 2035 [10]. These global ambitions acknowledge the importance of community involvement in the fight to end TB, particularly through strengthening the community health workforce.

The contribution of CHWs to TB care and prevention have been well documented [11, 12]. Specifically, CHWs have enabled the task-shifting of a variety of TB program responsibilities to optimize capacity utilization of the public health system. These decentralized tasks include active case finding [13]; sputum collection and transport, slide fixing and other laboratory support [14]; and treatment support, such as community-based directly observed treatment (DOT) and psychosocial support [15]. As a result, the deployment of CHWs in TB programs has been linked to improved patient treatment outcomes [16], increased case notification [17], and decreased treatment loss to follow-up (LTFU) [18]. Nevertheless, despite the substantial evidence on the activities and responsibilities that CHWs can assume, the understanding of best practices for engagement of CHWs and the operationalization of CHW models for TB care and prevention remains limited.

Stop TB Partnership established TB REACH in 2010 to foster innovative approaches and technologies in TB care and prevention and catalyze their scale-up. Over the course of eight waves, the TB REACH mechanism [19] has supported hundreds of projects utilizing numerous approaches to active case finding (ACF), many of which heeded the global call for greater involvement of CHWs. As such, TB REACH represents one of the world’s largest sources of funding and repository of information for interventions employing CHWs using a standardized monitoring and evaluation (M&E) framework [20].

## Objectives

The objective of this study was to evaluate TB REACH projects on how they identified, engaged, paid, trained, supervised and evaluated CHWs for the purposes of ACF and the impact of employing specific practices for CHW engagement on project performance.

## Methods

### Study design

This was a cross-sectional study analyzing secondary data from the TB REACH project repository and primary data from subsequent project implementers surveys. We followed the Strengthening the Reporting of Observational studies in Epidemiology (STROBE) checklist for cross-sectional studies (www.strobe-statement.org), which is available in Additional file 1: Table A7: STROBE Checklist.

### Project eligibility

We accessed the TB REACH Grant Management System (GMS) and included all projects from the first six TB REACH funding waves (*n* = 222) spanning from 2010 to 2020. We excluded projects unrelated to ACF, did not utilize CHWs, had invalid contact information, or did not include the primary TB REACH impact indicator of “additionality” in TB case notification [20]. In summary, this impact indicator is designed to capture the change in crude TB case notifications adjusted for trends in the pre-intervention period as well as in concurrent control areas.

### Data sources

#### TB REACH project data repository

We reviewed project summaries, quarterly narrative reports and annual grant reviews from the GMS for eligible projects. A data collection template with 24 key project characteristics was created on the ONA platform (ONA Systems, Nairobi, Kenya) for systematic information extraction and abstraction. We extracted project characteristics and standard indicators for the TB care cascade. The data were verified by research staff, adjusted for consistency, and compiled into a single dataset.

#### Project implementer survey

We developed a 54-question survey based on current published literature and WHO guidelines [4] to collect primary data on the CHW model employed by TB REACH grantees (see Additional file 1, Full Survey for Implementers). The survey instrument was provided through a web-based Google Forms survey and a Microsoft Word document. Survey flow and skip logic were built into the online survey and were clearly marked in the offline document. The full survey was sent to all primary contact persons of eligible projects. Secondary contacts were invited to complete the survey if the primary contact was unable to be reached or declined to participate. We requested participants to answer questions to the best of their ability and included an “unknown” response option to reduce recall bias. Anonymized data from the completed surveys were exported into Microsoft Excel. All survey responses were verified for completeness and consistency. The dataset was stored on a secure server and password protected with restricted access. To access the data, see Additional file 2.

### Data analyses

We calculated descriptive statistics for primary and secondary data. Additionality values from all projects included in the survey were summarized in a forest plot. We used a random-effects meta-analysis model to calculate the pooled additionality. We used the additionality indicator to trifurcate eligible projects into groups of low, medium, and high impact. The bottom, low-impact (LI) group achieved an additionality of ≤ 5%. The middle, medium-impact (MI) group consisted of projects that achieved an additionality of > 5% and ≤ 20%. The top, high-impact (HI) group reported an additionality of > 20%. Differences in responses for these three impact groups were assessed using the ANOVA or Kruskal–Wallis test for continuous variables and the Chi-squared or Fisher’s exact test for categorical variables. We fitted linear random-effect regressions to analyze the association between CHW factors and project additionality. We set the country of project implementation as the random effect. The primary outcome was the additionality indicator. The parameter coefficient (β̂’) was used to estimate the magnitude and direction of association. Univariate linear regressions were fitted between all possible CHW factors and additionality to identify significant factors to be included in the multivariate model (see Additional file 1: Table A1: Univariate association between community health worker activities and notification impact and Additional file 1: Table A2: Univariate association between community health worker factors and project additionality per community health worker (percent additionality trend adjusted)). Covariates with a *p*-value < 0.05 were included in the final model. Hypothesis tests were two-sided and point estimates included 95% confidence intervals (CI) for means and proportions, and inter-quartile ranges (IQR) for medians. Missing data or “unknown” responses were excluded from the analyses. Statistical analyses were performed on Stata v15 (StataCorp; College Station, TX, USA).

## Results

### Sample characteristics

**Fig. 1:**
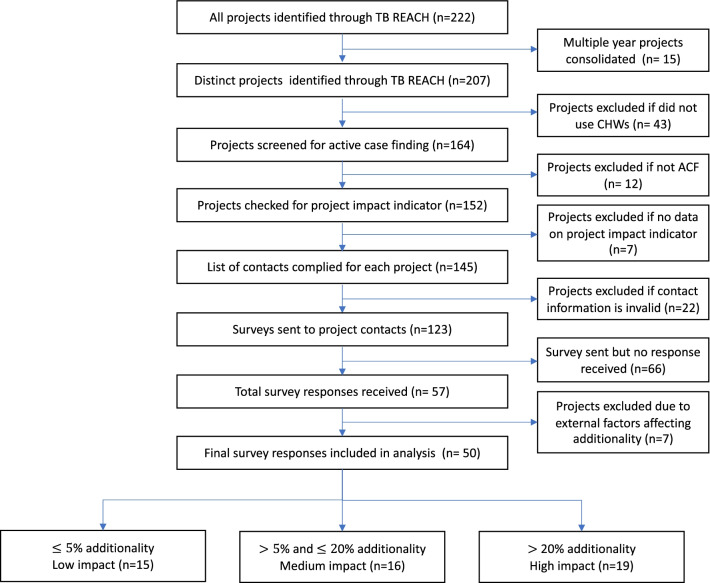
Project selection flowchart

**Fig. 2:**
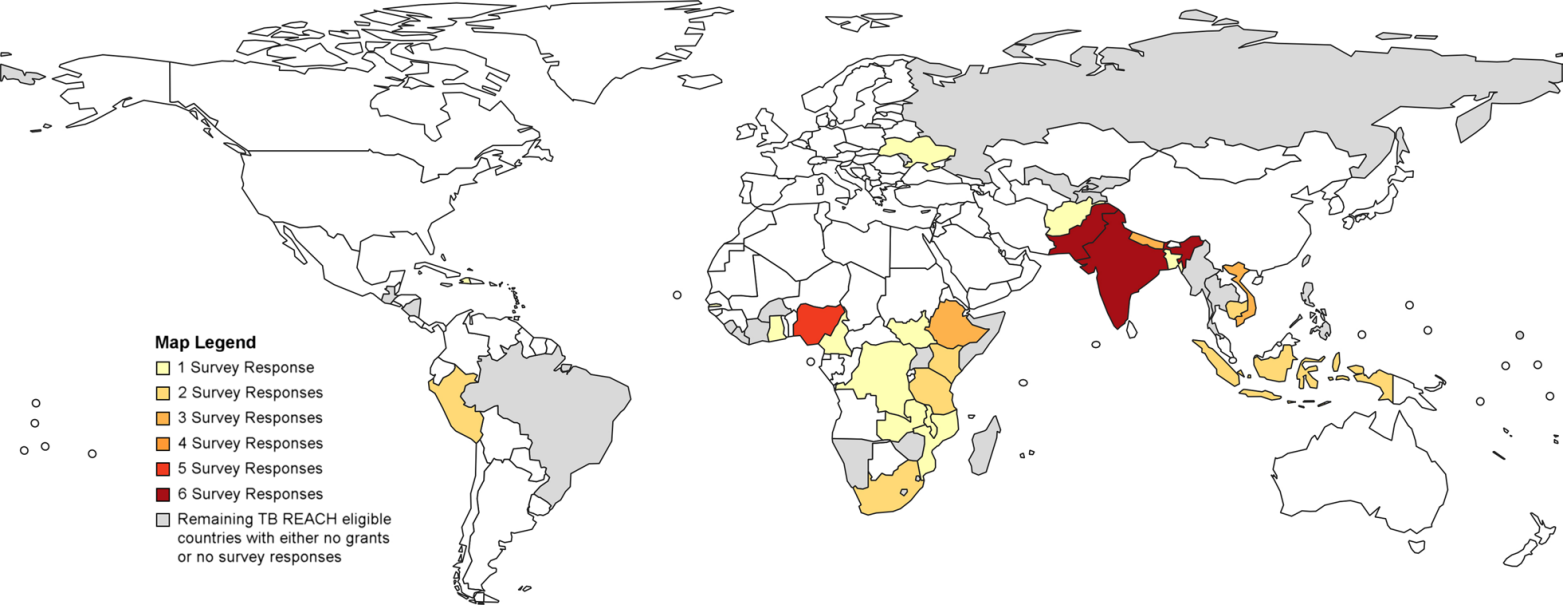
World map of TB REACH project countries

The TB REACH repository included 222 projects (Fig. 1). After excluding ineligible projects due to lack of CHW engagement (*n* = 43), no ACF activities (*n* = 12), and/or missing project impact indicator (*n* = 7), we identified 123 eligible projects for invitation to the implementer’s survey. We received a total of 57 survey responses over 6 weeks. Seven projects were excluded due to significant externalities confounding project additionality (see Additional file 1: Table A3: Factors Affecting Additionality) for a final sample of 50 projects from 24 countries (Fig. 2).

**Fig. 3:**
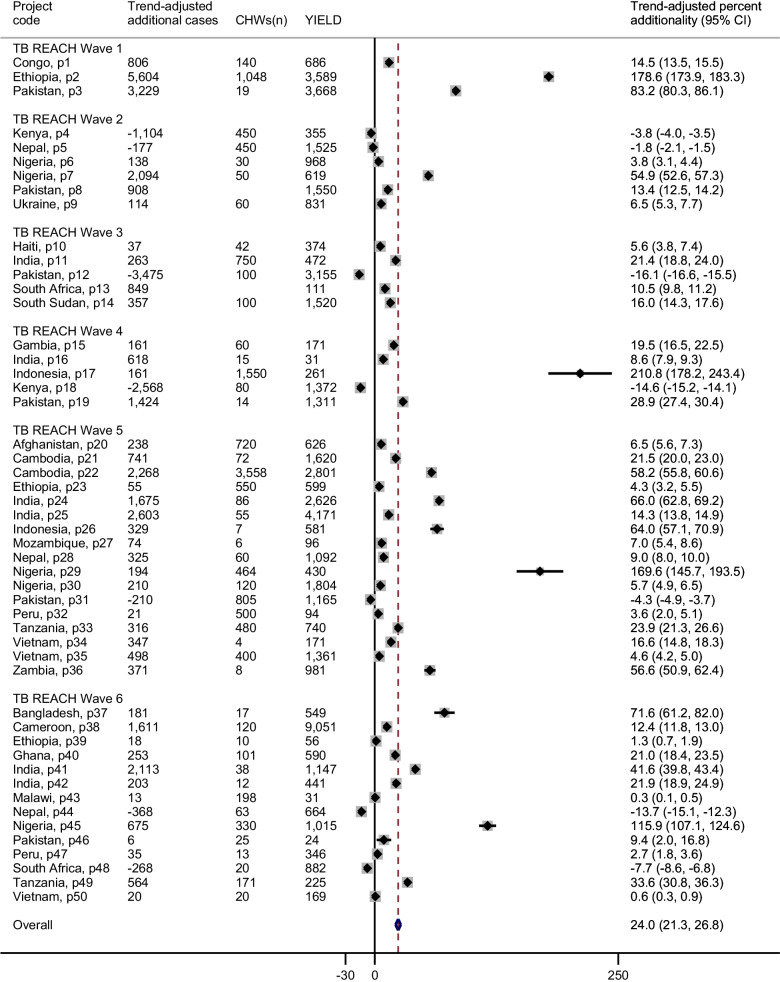
Forest plot of the additionality indicator

Pooled analysis provides a percentage additionality of 24.0% (95% CI 21.3–26.8%). The percentage additionality yield by projects ranged from -16.1% to 210.8% showing substantial heterogeneity (*I*^2^ = 99.9%) (Fig. **3**). When segmenting the sample by additionality into tertiles, we found an average additionality of − 2.7% for LI projects, 11.0% for MI projects, and 70.7% for HI projects (Table 1). The average grant duration was 19.4 months (95% CI 18.0–20.9) with an average value of $502 345 (95% CI $433 136—$571 553). The 50 projects employed a total of 13 991 CHWs with an average of 291 CHWs per project (range: 4–3558). These CHWs detected 58 717 TB cases, an average of 1174 per project. Of those detected, 19,481 would not have been identified without ACF activities through these projects. Approximately 68% of projects engaged CHWs to implement community-based active case finding, 6% employed CHWs in a health facility only, and 26% of projects had CHWs conduct activities in both settings. The high-impact group had the highest mean number of total CHWs employed (311). 66% of projects were implemented in the African and Eastern Mediterranean WHO region, and 68% (34/50) were conducted in lower-middle income countries (68%).

**Table 1:** Characteristics of TB REACH projects associated with notification impact

	LI (*n* = 15)	MI (*n* = 16)	HI (*n* = 19)	Total (*n* = 50)	p-value^╪^
% trend-adjusted additionality	0.3 (− 7.7–3.6)	10 (6.8–14.4)	56.6 (23.9–83.2)	12.9 (3.8–33.6)	< 0.001*
Average grant duration (months)	20.8	19.9	18.0	19.4	0.268
Average grant value (USD)	546 133	509 739	461 548	502 345	0.606
Type of project					
Community-based active case finding (ACF) only	13 (71%)	9 (69%)	12 (65%)	34 (68%)	0.353
Facility-based ACF only	0 (0%)	2 (13%)	1 (6%)	3 (6%)	
Community and facility-based ACF	2 (29%)	5 (19%)	6 (29%)	13 (26%)	
Peak number of community health workers (CHWs)^3^	70 (20–300)	55 (15–88)	86 (17.5–300)	60 (17.5–276)	0.354*
Total number of CHWs^2^	100 (20–450)	60 (25–120)	86 (17–86)	76 (20–425)	0.455*
Country regions [20]					
Africa	7 (48%)	6 (38%)	8 (42%)	21 (42%)	0.643
Americas	2 (13%)	1 (6%)	0 (0%)	3 (6%)	
South-East Asia	2 (13%)	4 (25%)	2 (10%)	8 (16%)	
Europe	0 (0%)	1 (6%)	0 (0%)	1 (2%)	
Eastern Mediterranean	2 (13%)	3 (19%)	7 (37%)	12 (24%)	
Western Pacific	2 (13%)	1 (6%)	2 (10%)	5 (10%)	
Project population	3,769,353	3,891,689	1,443,028	2,924,497	0.149
Tuberculosis care cascade					
Number of people verbally screened^3^	150 918 (24 629–430 025)	95,036 (10,116–335,536)	175,837 (91,093–451,628)	106 831 (33 322–365 601)	0.258*
Number of sputum samples collected^1^	7714 (3784—19 562)	6972 (1592–12 023)	5749 (3721–9705)	6,391 (3531–12 082)	0.443*
World Bank income classifications [21]					
Low	3 (20%)	6 (38%)	1 (5%)	10 (20%)	0.131
Lower-middle	9 (60%)	9 (56%)	16 (84%)	34 (68%)	
Upper-middle	3 (20%)	1 (6%)	2 (11%)	6 (12%)	

^1^: 1 (2%) respondent missing information on questions asked

^2^: 2 (4%) respondent missing information on questions asked

^3^: 5 (10%) respondent missing information on questions asked

^╪^: Fisher’s exact test and Chi-square tests: comparing proportions that is conditional on frequencies; ANOVA test: comparing means

^*^: Median and Kruskal–Wallis test

### CHW model characteristics

Table  2 presents key characteristics of CHW models. Most projects (62%) reported that CHWs only performed TB-related activities. For projects on which CHWs performed other tasks, CHWs spent an average of 80% of their time on TB-related activities. The majority of projects (86%) had CHWs complete community outreach and verbal screening. Forty-three projects (88%) provided CHWs with formal written contracts, and 33 (77%) of those projects provided all their CHWs with undifferentiated contracts. Almost all projects conducted training using experts (94%), while roughly half involved peers (54%). Classroom-based training was more commonly used than community-based training (96% vs 64%). Electronic distance learning was used only by a minority of projects exclusively in the HI group (11%). Thirty-one projects hosted periodic refresher trainings (65%).

**Table 2:** Key characteristics of the community health worker models associated with notification impact

	LI (*n* = 15)	MI (*n* = 16)	HI (*n* = 19)	Total	*p*-value^╪^
Implementation activities					
Tuberculosis (TB) and other	6 (40%)	5 (31%)	8 (42%)	19 (38%)	0.790
TB only	9 (60%)	11 (69%)	11 (58%)	31 (62%)	
% time spent on TB activities	77.3	85	77.9	80	0.649*
Community outreach^1^	13/15 (87%)	13/15 (87%)	16/19 (84%)	42/49 (86%)	1.00
Verbal screening^1^	13/15 (87%)	12/15 (80%)	17/19 (89%)	42/49 (86%)	0.730
HIV testing^1^	1/15 (7%)	1/15 (7%)	2/19 (11%)	4/49 (8%)	1.00
Sputum collection and transportation	12 (80%)	11 (69%)	13 (68%)	36 (72%)	0.712
Linkage to treatment	11 (73%)	9 (56%)	15 (79%)	35 (70%)	0.326
Treatment counseling	5 (33%)	7 (44%)	13 (68%)	25 (50%)	0.106
Recruitment and selection					
Had prior experience^2^	11/15 (73%)	10/14 (71%)	13/19 (68%)	34/48 (71%)	0.951
Years of education^3^	12 (10–14)	12 (10–12)	10 (9–12)	12 (10–12)	0.378*
Provided written contracts	13 (87%)	15 (94%)	15 (79%)	43 (86%)	0.462
From TB REACH	7 (54%)	13 (87%)	8 (53%)	27 (65%)	0.095
From non-governmental organization	4 (31%)	2 (13%)	4 (27%)	10 (23%)	0.513
From government	2 (15%)	1 (7%)	3 (20%)	6 (14%)	0.655
Provided differentiated contracts^5^	10/12 (83%)	14/15 (93%)	9/15 (60%)	33/42 (79%)	0.075
Pre-service training^¶^					
Training method					
Expert	14 (93%)	15 (94%)	18 (95%)	47 (94%)	1.000
Peer-to-peer	10 (67%)	8 (50%)	9 (47%)	27 (54%)	0.495
Hands-on	14 (93%)	13 (81%)	17 (90%)	44 (88%)	0.652
E-learning	0 (0%)	0 (0%)	2 (11%)	2 (4%)	0.323
Training setting					
Classroom-based	14 (93%)	15 (94%)	19 (100%)	48 (96%)	0.519
Community-based	8 (53%)	9 (56%)	15 (79%)	32 (64%)	0.223
Average hours of pre-service trainings^4^	12 (5–24)	12 (8–18)	16 (8–30)	16 (8–24)	0.366*
Refresher training^¶^					
Formal refresher trainings^2^	9/13 (69%)	11/16 (73%)	11/19 (58%)	31/48 (65%)	0.646
Formal training method (*n* = 31)					
Expert	8 (89%)	11 (100%)	10 (91%)	29 (94%)	0.740
Peer-to-peer	7 (78%)	10 (91%)	4 (36%)	21 (36%)	0.019
Hands-on	6 (67%)	10 (91%)	8 (73%)	24 (77%)	0.437
E-learning	0 (0%)	0 (0%)	2 (18%)	2 (7%)	0.314
Formal training setting					
Classroom-based	8 (89%)	10 (91%)	7 (64%)	25 (81%)	0.205
Community-based	4 (44%)	6 (55%)	7 (64%)	17 (55%)	0.692
Frequency of refresher trainings^4^	3 (1–4)	2 (2–4)	2 (2–4)	2 (2–4)	0.978*
Average hours of refresher trainings^3^	4 (3–8)	5 (3–8)	7 (3–8)	5.5 (3–8)	0.725*
Supervision					
Issues addressed by direct supervisor^4^	13/15 (87%)	13/13 (100%)	16/19 (84%)	42/47 (89%)	0.420
Issues addressed by upper management^4^	3/15 (20%)	0/13 (0%)	4/19 (22%)	7/47 (15%)	0.197
Female supervisor (%)^2^	58.8	53.5	29.5	45.6	0.007
Average # community health workers (CHWs) per supervisor^3^	13 (7–26)	7 (6–10)	15 (5–60)	9 (6–25)	0.247*
Average # of supervisor reviews per quarter^2^	9 (3–12)	6 (3–12)	6 (3–12)	6 (3–12)	0.775*
Average # of supervisor direct feedback per quarter^2^	9 (3–12)	3 (1–12)	6 (4–12)	6 (3–12)	0.293*
Sustainability and integration					
Promoted to a higher role	5/14 (36%)	8/13 (62%)	8/19 (42%)	21/46 (46%)	0.426
CHWs working on the project keep their jobs at the close of the project^2^					
All kept their jobs after project	7/14 (50%)	1/15 (7%)	5/19 (26%)	13/48 (27%)	0.064
A subset kept their jobs after project	4/14 (29%)	11/15 (73%)	11/19 (58%)	26/48 (54%)	
None kept their jobs after project	4/14 (29%)	2/15 (13%)	3/19 (16%)	9/48 (19%)	
Continued with the same responsibilities (*N* = 39)	8/11 (73%)	3/12 (25%)	13/16 (81%)	24/39 (62%)	0.007

Data are %, mean or median. % are calculated based on the total number of projects with available data. Percentages within each category are based on the total projects within each category. *N* sizes are listed for variables with missing values

^1^: 1 (2%) respondent missing information on questions asked

^2^: 2 (4%) respondents missing information on questions asked

^3^: 6 (12%) respondents missing information on questions asked

^4^: 3 (6%) respondents missing information on questions asked

^5^: 8 (16%) respondents missing information on questions asked

^╪^: Fisher’s exact test and Chi-square tests: comparing proportions that is conditional on frequencies; ANOVA test: comparing means

^*^: Median (IQR) and Kruskal–Wallis test

^¶^: As indicated by WHO CHW training guidelines, expert, peer-to-peer, and hands-on training indicates face-to-face interaction as opposed to distance learning (e-learning). Classroom-based training emphasizes theoretical knowledge; community-based training emphasizes practical application

Most projects cited that CHW performance issues were addressed by a direct supervisor (89%) rather than by upper management (15%). Projects employed slightly more male supervisors on average (54.4% male vs 45.6% female). Each supervisor oversaw an average of 33 CHWs, with a higher average in the high-impact group (60). All or a subset of CHWs kept their jobs after the TB REACH project ended in 81% of projects.

There were select notable differences between the three impact groups. There was an inverse relationship between the number of projects that provided their CHWs with the same contracts rather than differentiated labor agreements. We measured a higher proportion of projects using community-based training in the HI group, while lower impact projects completed more peer-to-peer training (see Additional file 1: Table A6: Differentiated contracts stratified by impact level). Projects in the HI category employed male supervisors significantly more frequently. Finally, there was a significant relationship between CHWs continuing with the same responsibilities after the close of a project and project impact (*p* = 0.007).

Table3 shows the results of the univariate and multivariate linear regression. Full results from the univariate analysis are listed in Additional file 1: Table A2: Univariate association between community health worker factors and project additionality per community health worker (percent additionality trend adjusted). Compared with projects that did not provide pre-service training through e-learning, projects that provided e-learning (*β̂* = 98.44, *p* < 0.001) were associated with higher additionality. An increase of one CHW per supervisor is associated with a 0.35 increase in the project’s additionality.

**Table 3:** Associations between community health worker factors and project’s trend-adjusted percent additionality

	Univariate analysis (*N* = 50)			Multivariate analysis (*N* = 41)		
	*n*	Coef. (95% CI)	*p*-value	*n*	Coef. (95% CI)	*p*-value
Peak number of CHWs	45	0.05 (0.01–0.09)	0.022	41	0.005 (− 0.04–0.05)	0.817
Total number of CHWs	48	0.03 (0.01–0.05)	0.013	41	0.008 (− 0.01–0.03)	0.482
Pre-service training—e-learning						
Yes	2	94.83 (29.65–160.01)	0.005	2	98.44 (44.82–152.06)	< 0.001
No	48	Ref		39		
Average CHWs per supervisor	44	0.38 (0.20–0.57)	< 0.001	41	0.35 (0.12–0.58)	0.002

### CHW compensation

Table 4 details compensation schemes employed on the TB REACH projects. Thirty-seven projects (74%) included or utilized a fixed component in their compensation, while 56% included or utilized variable compensation. The average total remuneration was US$ 153.31 per month. The average fixed component per month was US$ 124.44 and the average variable component per month was US$ 28.87. A breakdown of CHW compensation by income group and World Bank country income classification [22] is provided in Additional file 1: Table A4: CHW compensation by fixed and variable component and World Bank country classification [22]  and Additional file 1: Table A5: CHWs compensation by World Bank country classification.

**Table 4:** Monetary and non-monetary compensation of community health workers associated with notification impact

	LI (*n* = 15)	MI (*n* = 16)	HI (*n* = 19)	Total (*n* = 50)	*p*-value^╪^
Monetary compensation					
Earned only a fixed component (salary or stipend)	5 (33%)	6 (38%)	7 (37%)	18 (36%)	0.783
Earned only a variable component (incentives)^1^	3 (20%)	3 (19%)	2 (11%)	8 (16%)	
Earned both a fixed and variable component	5 (33%)	7 (44%)	7 (37%)	19 (38%)	
No monetary compensation	2 (13%)	0 (0%)	3 (16%)	5 (10%)	
Average total compensation/month (USD)^2^	127.77	210.62	127.51	153.31	0.106
Average fixed compensation/month (USD)	97.31	172.31	107.61	124.44	0.219
Average variable compensation/month (USD)	30.46	38.31	19.91	28.87	0.523
Non-monetary incentives^3^					
Priority access to TB/HIV or other disease testing	3/13 (23%)	4/12 (33%)	9/18 (50%)	16/43 (37%)	0.294
Health insurance	2/13 (15%)	1/11 (9%)	4/19 (22%)	7/43 (17%)	0.780

*N* sizes are listed for variables with missing values

^1^: 2 (4%) respondent missing information on questions asked

^2^: 8 (16%) respondent missing information on questions asked

^3^: 7 (14%) respondent missing information on questions asked

^**╪**^Fisher’s exact test and Chi-square tests: comparing proportions that is conditional on frequencies; ANOVA test: comparing means

There was no significant difference in total, fixed or variable compensation across the three impact groups. The greatest average total compensation per month was provided by the MI group (US$ 201.62); the total compensation by LI and HI groups were comparable (US$ 127.77 and US$ 153.32, respectively). There was a larger proportion of projects from the HI and MI groups where CHWs were given priority access to disease testing (*p* = 0.294).

## Discussion

Our retrospective data collection and analysis of 50 survey responses from TB REACH project implementers provided information on a broad range of CHW engagement models for TB interventions carried out globally. This information contributes to filling the knowledge gaps in the existing literature on the roles of CHWs and elucidates variables facilitating project success. The results of our study found that CHW interventions overall had a large positive impact on yielding incremental TB notifications in intervention areas. The CHW model factors more likely to influence project outcomes are the training method and setting, supervisory models, and integration into the existing health system.

Regarding training, we found capacity building through hands-on or practical applications in a community setting was associated with more successful case finding. This was concordant with a systematic review that showed that outcomes are improved when training is combined with other capacity building measures, and an additional review that found that training for diagnostic tests should be addressed through hands-on practice and consistent feedback from experienced health care workers or supervisors [23, 24]. Training completed in the community is also highlighted by Gilroy and Winch, in which a commonly cited problem in CHW studies is “training that is too classroom-based with little practical hands-on experience” [25]. While our survey found that the classroom was the most common training setting, some projects reported training in both the classroom and the community. The higher impact groups had a larger proportion of projects that incorporated community-based training. This reinforces that training CHWs on practical skills in the community which they will eventually serve can contribute to better prepared health workers.

Our multivariate regression found that the two projects utilizing e-learning methods during pre-service training yielded higher additionality. Although e-learning is not yet commonly used for CHW training, the current COVID-19 pandemic may catalyze a potentially positive paradigm shift in capacity building. A study in sub-Saharan Africa found that the blended approach combining e-learning with classroom training for CHWs improved standardization of training content and retention to course materials due to the ability to revisit online course materials [26]. The study also showed a total training cost savings of 42% from reduced travel, personnel, and classroom costs. Thus, e-learning proves to be a promising mode of training. Further studies and projects should explore the potential of integrating mobile or remote health technologies in their training models, especially given the new realities of travel and meetings in many countries.

The majority of projects in our survey had CHW performance issues addressed by a direct supervisor without the need of upper management involvement. Supervisors provided direct feedback to CHWs an average of 13 times per quarter (3 months), leading us to believe that the regularity of feedback is unlikely to influence project outcomes. However, a previous study on CHW models have also noted that regular supervision can be demotivating as some CHWs perceive their supervision to be linked to their poor performance [27]. Conversely, more supportive supervision by formal health workers can provide CHWs with a sense of legitimacy and increased work motivation [28]. As the evidence base remains ambiguous, more research is needed on the most optimal roles of supervisors should play in order to best support CHWs.

The effective integration of CHWs into the existing health systems is a well-documented enabler of project success [5, 6] as it enhances sustainability and fosters collaboration between lay and formal healthcare providers. CHWs have also vociferously noted an enhanced credibility and clarity of their own roles with a greater level of integration [5, 29]. Our study results were concordant with these findings. Projects that had CHWs continue the same roles and responsibilities after the close of the project tended to produce a greater additionality impact on TB notifications. This may suggest that projects offering the prospect of long-term engagement through ties with the public health system and other health networks had a positive impact on CHW performance. Another hypothesis is that CHWs originally employed by the project decided to continue their roles because the project was successful. Pallas et al. posit that integrating CHW programs into the agendas of ministries of health, non-governmental organizations, and international donors can create more impactful CHW models that are sustainable even during times of political unrest or a reduction of external donor funding [30]. Thus, it is beneficial to embed CHW models within primary health care systems for them to be both sustainable and impactful.

More projects that we surveyed compensated their CHWs through a fixed component (salary or stipend) than through a variable component. A study on three large CHW programs in India has found an unmet demand for salaried positions and service conditions for CHWs and that the volunteer status can hinder CHWs from receiving job benefits and fair salaries. This study specified that performance-based incentives lack financial security in large-scale CHW programs such as the Accredited Social Health Activist (ASHA) [31]. Moreover, our survey also evinced that more projects in the high-impact group tended to provide their CHWs with additional benefits beyond monetary compensation, including the provision of health insurance and priority access to disease testing. This highlights that additional benefits contribute to higher CHW job satisfaction and performance, which in turn may translate into to higher outputs and larger impact of the project. The importance of fair and commensurate compensation and the potentially positive impact on TB case notifications has been noted in other studies as well [32].

A key strength of this study was the unique data source. As one of the largest funding mechanisms for TB, our access to the GMS allowed us to use a robust dataset on the characteristics and outcomes of projects with CHW engagement models across many different countries. Access to this database also facilitated the recruitment of project implementers. Our survey was developed to closely align with the WHO guidelines on health policy and system support to optimize CHW programs, allowing us to obtain details on how concrete implementers’ experiences support or discount the recommendations of these guidelines, and which of the recommendations to prioritize in the context of leveraging CHWs for TB ACF. As such, while our study results are largely concordant with WHO recommendations, concrete experiences from TB REACH projects utilizing CHWs identified factors such as community-based training, rigorous support and supervision, and intentional integration into the existing health care system that may be prioritized to increase the likelihood of incremental notification impact and successful implementation of CHW models for TB.

Our study was limited by the attempt to associate project and CHW characteristics with TB REACH’s primary outcome metric, trend-adjusted changes in TB case notifications (additionality). While this can offer a less biased measure of performance, we had to exclude a number of projects for which the additionality assessment was confounded by external factors (see Additional file 1: Table A3: Factors Affecting Additionality). Additionality is also only one metric by which active TB case finding interventions can be assessed, and it is possible that projects that yielded a low additionality could have had a high impact on other evaluation metrics. We also experienced a low overall response rate (41%) to our survey. Several implementers noted that responses to specific survey questions may not be accurate due to difficulties recalling details from projects implemented many years ago.

## Conclusion

Interventions that use CHWs documented large increases in the number of people treated for TB in project areas. This study elucidated possible factors that have a larger impact on TB ACF projects using CHWs. In line with the 2018 WHO guidelines for CHW models, this study highlights that CHWs should be provided with ongoing dynamic types of training, supportive and continuous supervision, and fair compensation as well as be well-integrated into the existing primary healthcare system. These areas may be prioritized when designing TB projects with a CHW engagement component in order to enhance the potential for a higher notification impact and more sustainable engagement of CHWs.

## Supplementary Information

**Additional File 1. Tables A1–A7 and Full Survey for Implementers.** Tables include the Univariate association between community health worker activities and notification impact, Uni variate association between community health worker factors and project additionality per community health worker (percent additionality trend adjusted), Factors Affecting Additionality, CHW compensation by fixed and variable component and World Bank country classification, CHWs compensation by World Bank country classification, Differentiated contracts stratified by impact level, and STROBE Checklist.

**Additional File 2.** **Data from TB REACH project data repository and project implementer survey.** Data mined from TB REACH project data repository combined with the project implementer survey.

## Data Availability

All data generated or analyzed during this study are included in this published article and its supplementary information files.

## Abbreviations

ACF: Active case finding
CHW: Community Healthcare Worker
DOT: Directly observed treatment
GMS: Grant management system
HI: High-impact
HIV: Human immunodeficiency virus
IQR: Inter-quartile ranges
LI: Low-impact
LTFU: Loss to follow-up
M&E: Monitoring and evaluation
MI: Medium-impact
STROBE: Strengthening the Reporting of Observational studies in Epidemiology
TB: Tuberculosis
WHO: World Health Organization
