# Supplementary material for: What makes community health worker models for tuberculosis active case finding work? A cross-sectional study of TB REACH projects to identify success factors for increasing case notifications
Source: Hum Resour Health. 2022 Mar 12;20:25. doi: 10.1186/s12960-022-00708-1 (PMC8917377; doi:10.1186/s12960-022-00708-1)
Supplement: Supplementary file 1 — Additional File 1. Tables A1–A7 and Full Survey for Implementers. Tables include the Univariate association between community health worker activities and notification impact, Uni variate association between community health worker factors and project additionality per community health worker (percent additionality trend adjusted), Factors Affecting Additionality, CHW compensation by fixed and variable component and World Bank country classification, CHWs compensation by World Bank country classification, Differentiated contracts stratified by impact level, and STROBE Checklist. [file 12960_2022_708_MOESM1_ESM.pdf]

Table A1: Univariate association between community health worker activities and notification impact

	Coef.	95%CI	p-value
TB and other activities	ref	··	··
Solely TB	-14·73	-42·97 – 13·51	0·300
Time spent on TB-related activities	-0·34	-0·76 – 0·77	0·107
Community outreach	-0·62	-41·05 – 39·80	0·975
Verbal screening	18·41	-21·65 – 58·48	0·360
HIV testing	-7·44	-59·06 – 44·19	0·733
Sputum collection and transportation	-5·11	-35·95 – 25·74	0·741
Linkage to treatment	10·23	-19·87 – 40·34	0·498
Treatment counseling	20·94	-6·11 – 48·00	0·126

Table A2: Univariate association between community health worker factors and project additionality per community health worker (percent additionality trend adjusted)

	Coef.	95%CI	p-value
**CHW characteristics**			
Age	-0·99	-2·87 -0·90	0·296
Sex			
Male (%)	-0·15	-0·66 – 0·35	0·543
Female (%)	0·20	-0·29 – 0·69	0·425
Other (%)	-0·33	-1·59 – 0·93	0·599
Prior experience	-2·89	-34·56 – 28·77	0·855
Years of education	-1·10	-5·27 -3·07	0·597
At the height of the project, number of CHWs employed in one month	0·05	0·01 – 0·09	**0·022**
During the full duration of the project, number of CHWs on the project	0·03	0·01 – 0·05	**0·013**
CHWs peak/total	0·11	-0·25 – 0·49	0·527
**Recruitment/selection**			
CHWs provided with written contracts	-28·14	-67·26 – 10·97	0·154
Contracts provided			
By TB REACH grantee	-17·71	-45·09 – 9·67	0·199
By a government entity	27·25	-10·21 – 64·72	0·149
An NGO (not grantee)	2·95	-28·56 – 34·46	0·851
All CHWs provided the same contract	-29·97	-59·86 – 0·08	**0·049**
**Training**			
**Pre-service training**			
**Type of pre-service training**			
Face-to-face with expert	6·11	-52·24 - 64·46	0·834
Classroom	23·08	-47·36 - 93·51	0·513
Community	19·28	-9·06 - 47·61	0·178
Peer-to-peer	-10·90	-38·54 - 16·73	0·432
E-learning	94·83	29·65 - 160·01	**0·005**
Hands on	18·02	-24·32 - 60·37	0·396
Pre-service training hours	0·59	-0·38 - 1·56	0·229
Training compensation provided	-33·00	-74·84 - 8·84	0·119
Training materials were provided to the CHWs during training	-4·33	-47·78 - 39·11	0·842
Training certificate	4·82	-26·23 - 35·87	0·756
**Preservice training: Treatment support**			
DOT procedures	28·86	-10·97 - 68·68	0·151
Patient counselling	14·41	-28·54 - 57·36	0·503
Social and psychological needs of TB patients	-22·63	-54·86 - 9·60	0·164
Psychosocial support	-0·61	-34·32 - 33·10	0·971
**Refresher training**			
Frequency of refresher training	0·67	-2·47 - 3·82	0·668
Average length of refresher training	0·58	-1·15 - 2·29	0·504
**Types of refresher training**			
Formal	6·51	-23·45 - 36·47	0·664
Informal/None	ref	··	··
**Formal Training**			
Face-to-face with expert trainer	12·94	-70·89 - 96·77	0·754
Face-to-face in classroom	-32·74	-83·46 - 17·96	0·197
Face-to-face in community	21·71	-18·91 - 62·34	0·283
Peer-to-peer training	-31·71	-74·16 - 10·74	0·137
E-learning	77·54	-1·09 - 156·18	0·053
Hands on training	26·05	-22·29 - 74·38	0·279
Training compensation provided	12·34	-15·01 - 39·70	0·368
**Compensation**			
**Fixed component (salary or stipend)**			
Yes	-5·16	-36·74 - 26·41	0·744
**Compensation was constant**			

Yes (constant)	9·54	-22·65 - 41·73	0·551
No (changed)	ref	··	··
**CHWs compensated equally for the fixed component, or did it vary within implementation areas**			
Compensated equally	ref		
Compensated differently	9·30	-23·17 - 41·78	0·564
Average compensation/month	12·34	-15·01 - 39·70	0·368
**CHW compensation - variable components**			
Variable component (performance-based incentives)	8·49	-15·79 - 32·78	0·485
Average compensation/month	-0·04	-0·37 - 0·27	0·755
Average minimum compensation/month	-0·48	-1·85 - 0·89	0·475
Average maximum compensation/month	-0·13	-0·40 - 0·13	0·303
**CHW compensation - non-monetary components**			
Food	-15·90	-58·82 - 27·03	0·459
Supplements	-14·64	-64·21 - 34·91	0·554
Stationery	21·15	-24·41 - 66·71	0·354
Clothing	12·68	-19·70 - 45·07	0·434
Mode of transport (motorbike, car, bicycle)	5·41	-26·67 - 37·49	0·735
Right to retain project technology such as tablets or phones	3·88	-29·11 - 36·86	0·814
Priority access to TB/HIV or other disease testing	-9·09	-41·98 - 23·78	0·580
Health insurance	-11·82	-54·88 - 31·24	0·582
**Supervision**			
Performance issues addressed by direct supervisor	-1·40	-49·18 - 46·39	0·953
Performance managed addressed by upper management	19·84	-21·11 - 60·80	0·334
**Gender of Supervisors**			
Male (%)	0·41	-0·06 - 0·88	0·086
Female (%)	-0·41	-0·88 - 0·07	0·086
Average CHWs per supervisor	0·38	0·20 - 0·57	**<0·001**
**Paper- or electronic-based tools used to monitor CHW activities**			
Paper based	ref		
Electronic	-10·33	-63·62 - 42·93	0·698
Both	-4·04	-33·33 - 25·24	0·782
**Specific tools of screening**			
Screening data	5·32	-53·04 - 63·69	0·855
GPS data	-15·65	-61·64 - 30·34	0·497
Timesheets	-12·77	-40·82 - 15·29	0·365
Financial data	0·94	-29·93 - 31·82	0·951
Verification with external stakeholders (clinic staff)	-6·41	-34·44 - 21·62	0·648
Lab registers	9·86	-18·08 - 37·81	0·481
Routine surveillance data	9·50	-19·64 - 38·63	0·515
Customer Relationship Management Software	-34·73	-92·23 - 22·77	0·231
Times supervisor reviewed work of CHWs per quarter	-0·04	-0·80 - 0·71	0·912
Times supervisor provided direct feedback per quarter	-0·04	-0·67 - 0·58	0·876
**Mobility and Sustainability**			
**CHWs promoted to a higher role within your organization during or after implementation of the project**			
Yes	-20·52	-50·02 - 8·99	0·168
**CHWs working on the TB REACH project keep their jobs at the close of the project?**			
All CHWs kept their jobs after project	12·14	-31·49 - 55·76	0·578
A subset of CHWs kept their jobs after project	11·05	-27·86 - 49·96	0·570
No	ref		
**CHWs continue working with the same roles and responsibilities as during the TB REACH grant?**			
Yes	31·41	-2·71 - 65·53	0·070

Table A3: Factors Affecting Additionality

Additionality	Reason	Recommendation	N baseline
**Top 20% additionality projects**			
2617	Dilution risk due to small target population spread out over a large geographical area. Project did not have a control population. Additionality is calculated based on self-reported TB history.	Exclude	23
1033	Historical baseline case numbers were very small (4 and 7), causing additionality to reach a value over 1000%.	Exclude	1
211	211% unadjusted change in baseline was calculated based on unusually low baseline data from facility registers. Project suggested using an alternative 59% additionality. This was the value utilized in our study.	Use recommended 59% additionality	27
206	Trend adjusted increase explained by an exponential rise in pediatric TB case detection in all intervention hospitals.	Include	92
156	Upward progress in notifications due to project activity.	Include	28
138	Improvements of case detection due to project efforts to enhance access to X-rays by providing transportation incentives.	Include	106
93·8	No external activities or interacting factors to explain the increase in the number of cases in the intervention area.	Include	762
82·7	No external factors cited for increase in case finding.	Include	468
66	Decline in notifications in evaluation population due to NGO stopping ACF in 3 out of 4 areas, however there was a general decline in notifications. All forms cases are likely to be additional.	Include	505
64	Lower case rates in the control population, making the case rates less comparable to the evaluation population.	Include	124
58	Clear increase from baseline that is higher than the control population.	Include	1001
**Bottom 20% additionality projects**			
-100	Value from baseline report. Overall project additionality could not be found.	Exclude	1367
-22	Unstable and delayed notification since data only available until 2015.	Exclude	10653
-17·75	Change from paper-based to web-based reporting, potentially contributing to incomplete notifications.	Exclude	127
-10	Overlapping projects, confounding effects from prior and concurrent projects.	Exclude	1924
-7	External factors included health care workers on strike and national elections closing public health facilities, poor sampling by community volunteers, weak lab capacity, and only 40% of health facilities reported notifications for the first 2 quarters.	Exclude	660
-6	Unadjusted additionality for Bac+ is positive, All Forms lower than predicted.	Include	1127
-4	Decline in notifications by 4%, noted high level of seasonality.	Include	1209
-3·8	Hypothesis for negative additionality is that some case finding work in the region was already in effect before the project started.	Include	5397
-1	Some cases detected not shown in metrics due to open screening initiative.	Include	338
1	Lower additionality due to delays in final project permissions.	Include	714
1	Historical notification data shows a decline in Bac+ notifications.	Include	528

Table A4: CHW compensation by fixed and variable component and World Bank country classification

	LI (n=15)	MI (n=16)	HI (n=19)	Total (n=50)	p-value
**Total compensation (Variable + fixed)**					
Average total compensation/month (USD)	127·77	210·62	127·51	153·31	0·106
Low income countries	118·75	50·00	45·33	89·85	··
Lower middle income countries	132·20	172·00	141·93	149·23	··
Upper-middle income countries	301·50	400·00	··	334·33	··
**Fixed-component compensation**					
Earned a fixed component (salary or stipend)	10 (67%)	13 (81%)	14 (74%)	37 (74%)	0·651
Average fixed compensation/month (USD)	97·31	172·31	107·61	124·44	0·219
Low income countries	125·00	50·00	11·67	62·14	··
Lower middle income countries	101·60	131·36	118·42	117·61	··
Upper-middle income countries	301·5	400·00	··	334·33	··
**Variable-component compensation**					
Earned a variable component (incentives)	8 (57%)	10 (63%)	9 (50%)	27 (56%)	0·762
Average variable compensation/month (USD)	30·46	38·31	19·91	28·87	0·523
Low income countries	33·33	0·00	33·67	27·71	··
Lower middle income countries	30·60	40·64	23·51	31·61	··
Upper-middle income countries	0·00	0·00	··	0·00	··
**Non-monetary incentives**					
Priority access to TB/HIV or other disease testing	3 (23%)	4 (33%)	9 (50%)	16 (37%)	0·294
Health insurance	2 (15·4%)	1 (8·3%)	4 (22·2%)	7 (16%)	0·597
**GDP per capita (USD)**					
Average GDP per capita (USD)	2539·23	1757·31	1983·35	2077·78	0·368
Low income countries	565·75	719·10	909·50	714·88	··
Lower middle income countries	1737·54	2067·48	1693·82	1838·56	··
Upper-middle income countries	6977·70	6652·27	4135·60	5704·90	··

Table A5: CHWs compensation by World Bank country classification

	Low Income	Lower-middle Income	Upper-middle Income
Provided fixed compensation	6 (60%)	28 (82%)	3 (50%)
Average fixed compensation/month (USD)	92·00	139·39	334·33
Fixed income as a % of GDP/capita	16·6%	7·8%	5·3%
Provided variable compensation	6 (60%)	21 (62%)	0 (0%)
Average variable compensation/month (USD)	50·25	51·35	··
Variable income as a % of GDP/capita	7·8%	3·3%	··

Table A6: Differentiated contracts stratified by impact level

	LI	MI	HI	Total
**Provided different contracts**	1	2	7	10
Different contracts for salaried vs volunteer workers	1	1	0	2
Different contracts based on position	0	0	1	1
Different contracts from NGO vs. government or other partners	0	0	4	4
Additional transportation contract	0	0	1	1
Incentive based contracts	0	0	1	1
Unknown	0	1	0	1

Table A7: STROBE Checklist

Section	Recommendation	Comments
Title	Indicate the study's design with a commonly used term in the title or the abstract	Completed
Abstract	Provide in the abstract an informative and balanced summary of what was done and what was found	Completed
Introduction – background/rationale	Explain the scientific background and rationale for the investigation being reported	Completed
Introduction – objectives	State specific objectives, including any prespecified hypotheses	Completed
Methods – study design	Present key elements of study design early in the paper	Completed
Methods – setting	Describe the setting, locations, and relevant dates, including periods of recruitment, exposure, follow-up, and data collection	Completed
Methods – participants	Give the eligibility criteria, and the sources and methods of selection of participants	Completed
Methods – variables	Clearly define all outcomes, exposures, predictors, potential confounders, and effect modifiers. Give diagnostic criteria, if applicable	Diagnostic criteria not relevant to this study
Methods – data sources/measurement	For each variable of interest, give sources of data and details of methods of assessment (measurement). Describe comparability of assessment methods if there is more than one group	Full survey provided in supplemental files.
Methods – bias	Describe any efforts to address potential sources of bias	Completed
Methods – study size	Explain how the study size was arrived at	Completed
Methods – quantitative variables	Explain how quantitative variables were handled in the analyses. If applicable, describe which groupings were chosen and why	Completed
Methods – statistical methods	(a) Describe all statistical methods, including those used to control for confounding<br>(b) Describe any methods used to examine subgroups and interactions<br>(c) Explain how missing data were addressed<br>(d) If applicable, describe analytical methods taking account of sampling strategy<br>(e) Describe any sensitivity analyses	Completed, (e) – sensitivity analyses not applicable
Results - participants	(a) Report numbers of individuals at each stage of study—e.g. numbers potentially eligible, examined for eligibility, confirmed eligible, included in the study, completing follow-up, and analyzed<br>(b) Give reasons for non-participation at each stage<br>(c) Consider use of a flow diagram	Completed
Results – descriptive data	(a) Give characteristics of study participants (e.g. demographic, clinical, social) and information on exposures and potential confounders<br>(b) Indicate number of participants with missing data for each variable of interest	Completed
Results – outcome data	Report numbers of outcome events or summary measures	Completed
Results – main results	(a) Give unadjusted estimates and, if applicable, confounder-adjusted estimates and their precision (e.g., 95% confidence interval). Make clear which confounders were adjusted for and why they were included<br>(b) Report category boundaries when continuous variables were categorized<br>(c) If relevant, consider translating estimates of relative risk into absolute risk for a meaningful time period	(a) Completed<br>(b) – continuous variables were not categorized in this study<br>(c) – relative risk not relevant to this study
Results – other analyses	Report other analyses done—e.g. analyses of subgroups and interactions, and sensitivity analyses	Univariate and multivariate linear regression completed. Other analyses included in thesupplemental files
Discussion – key results	Summarize key results with reference to study objectives	Completed
Discussion – limitations	Discuss limitations of the study, taking into account sources of potential bias or imprecision. Discuss both direction and magnitude of any potential bias	Completed
Discussion – interpretation	Give a cautious overall interpretation of results considering objectives, limitations, multiplicity of analyses, results from similar studies, and other relevant evidence	Completed
Discussion – generalizability	Discuss the generalizability (external validity) of the study results	Completed
Other information – funding	Give the source of funding and the role of the funders for the present study and, if applicable, for the original study on which the present article is based	Completed

Full Survey for Implementers

Thank you for your participation in our study about the use of healthcare workers in your active case finding project. The study will help us gain insight into the factors and systems that enabled projects to successfully engage community healthcare workers (CHWs).

We are using the term CHW broadly in this survey since in TB REACH Waves 1 through 6, 157 projects from 42 different countries used some form of community layperson to conduct TB active case finding. Your project may have called this cadre of worker another name than CHW such as lady healthcare workers, TB screeners, or ASHAS, but for the purpose of this survey, we are grouping all of these workers under the name CHW.

Please submit this Word document by email at chwstudy@tbhelp.org. If you have already filled out the Google Form survey, you do not need to send this document.

1. Organization name:	2. Primary contact name:
3. Email of primary contact:	4. Job title of primary contact:
5. Country:	

General project information:

6. Title of the TB REACH project	
7. Project Code	
8. Type of project (check all that apply)	☐ Community-based ACF<br>☐ Facility-based ACF<br>☐ Private sector<br>☐ Public sector
9. Did the project employ CHWs to conduct TB active case finding?	☐ Yes ☐ **No* If no, please do not continue to fill out this survey.**

Information about CHWs working in the project

10. What was the average age of a CHW in your project?	____________years
11. What was the gender breakdown of the CHWs employed in the project?	______% Male<br>______% Female<br>______% Other<br><br>Please specify how you define "Other":
12. Was any prior work experience/ training / certification required for employment in the project?	☐Yes ☐No ☐Unknown<br>12a. If yes, what type of work experience/training/certification?
13. On average, how many years of education did the CHWs have?	____________years
14. At the height of your project, how many CHWs were employed in one month?	
15. During the full duration of the project, how many CHWs worked on the project?	
16. Did the CHWs engaged in your project solely work on TB-related activities or were they part of a larger program that performed other health-related activities (such as maternal and	☐Solely TB-related activities<br>☐TB and other health-related activities<br>16a. If TB and other health-related activities, what other tasks were the CHWs asked to complete?

child health, vaccines, HIV, population control etc.)?	16b. If TB and other health-related activities, what was the estimated percentage of their time (FTE) spent on TB-related work:<br>___%

Selection of CHWs

17. What were the main sources you used to find potential CHWs? (For example, CHWs were sourced from volunteers at the district hospital or from an existing pool of HIV counsellors)	
18. Were interviews conducted to select CHWs in your project?	☐Yes ☐No ☐Unknown

Contracting of CHWs

19. Were CHWs provided with written contracts?	☐Yes ☐No ☐Unknown
20. Who provided the contract?	☐ The TB REACH grantee<br>☐ A government entity<br>☐ An NGO (not the grantee)<br>☐ Other_______________
21. Did all CHWs receive the same contract?	☐Yes, all received the same contract<br>☐No, the project had more than one type of CHW contract
	21a. If No, please describe the types of contracts provided to CHWs:

Pre-service Training of CHWs

22. What type of pre-service training was conducted? ***(check all that apply)***	☐ Face-to-face with an expert trainer in a classroom setting<br>☐ Face-to-face with an expert trainer in a community setting<br>☐ Peer-to-peer training in a classroom setting<br>☐ Peer-to-peer training in a community setting<br>☐ e-learning<br>☐ Hands on practice of skills in a classroom setting<br>☐ Hands on practice of skills in a community setting<br>☐ Other _____________
23. On average, for how many hours was the pre-service training of a CHW?	______ hours
24. Was training mandatory for all CHWs working in the project?	☐Yes ☐No ☐Unknown
25. Were CHWs compensated for participating in the training?	☐Yes ☐No ☐Unknown
	25a. If yes, check the types of compensation:<br>☐ Cash per diem<br>☐ Food during training<br>☐ Other _____________
26. Which types of training materials were provided to the CHWs during training? ***(Check all that apply)***	☐ Project-specific handouts<br>☐ Project- specific diagnostic algorithms<br>☐ Project standard operating procedures (SOPs)<br>☐ Published scientific research<br>☐ NTP guidelines<br>☐ None

27. Was a certificate provided upon completion of the training?	☐Yes ☐No ☐Unknown
	27a. If yes, who issued the certificate?

Please select the topics that CHWs in your project were trained on prior to implementation of the grant:

**28. TB Information**	
TB disease and pathogenesis	☐Yes ☐No ☐Unknown
Airborne infection control & personal safety measures	☐Yes ☐No ☐Unknown
Bacteriological diagnosis of TB	☐Yes ☐No ☐Unknown
Clinical diagnosis of TB	☐Yes ☐No ☐Unknown
NTP guidelines	☐Yes ☐No ☐Unknown
Patient confidentiality	☐Yes ☐No ☐Unknown
**29. TB Screening**	
Screening procedures	☐Yes ☐No ☐Unknown
Data collection on paper	☐Yes ☐No ☐Unknown
Electronic data collection	☐Yes ☐No ☐Unknown
Methods for contact tracing	☐Yes ☐No ☐Unknown
Community mobilization strategies	☐Yes ☐No ☐Unknown
**30. Managing sputum**	
Coaching on how to produce a sputum sample	☐Yes ☐No ☐Unknown
Sample collection by CHWs	☐Yes ☐No ☐Unknown
Sample transport guidelines	☐Yes ☐No ☐Unknown
Sample storage guidelines	☐Yes ☐No ☐Unknown
**31. Treatment support**	
DOT procedures	☐Yes ☐No ☐Unknown
Patient counselling	☐Yes ☐No ☐Unknown
Social and psychological needs of TB patients	☐Yes ☐No ☐Unknown
How to provide psychosocial support	☐Yes ☐No ☐Unknown
**32. Project-specific information**	
Expected roles and responsibilities	☐Yes ☐No ☐Unknown
Collaboration with other CHWs	☐Yes ☐No ☐Unknown
Integration of TB services into the wider healthcare system	☐Yes ☐No ☐Unknown
33. Please list any other topics that CHWs were trained on as part of their pre-service training:	

The next section asks about various components of the compensation package provided to CHWs including, fixed components (such as salaries), variable components (like performance-based incentives), and non-monetary components.

Compensation- fixed components

34. Did the CHWs earn a fixed component (such as a salary or stipend) per month?	☐Yes ☐No* ☐Unknown*<br>***If No or Unknown please skip to the next section on variable components**
34a. Did the fixed compensation package change over the duration of the grant?	☐ The same compensation was offered throughout the entire project<br>☐ Compensation changed over time

	34b. If compensation changed over time, how did it change? Why was it changed?
34c. Were all CHWs compensated equally for the fixed component or did it vary within the implementation areas? (Perhaps it differed for CHWs in urban vs. rural areas or because of specific training or certification, or because CHWs were asked to complete different tasks)	☐ The project paid equal, fixed compensation to each CHW<br>☐ The fixed compensation differed for some CHWs
	34d. If fixed compensation varied, how did it vary?
34e. On average, how much fixed compensation did CHWs earn per month in the project?	________ USD

Compensation- variable components

35. Did the CHWs earn a variable component (such as performance-based incentives) per month?	☐Yes ☐No***** ☐Unknown*****<br>****If No or Unknown please skip to the next section on non-monetary compensation***
35a. Did the variable compensation package change over the duration of the grant?	☐The same compensation was offered throughout the entire project<br>☐ Variable compensation changed over time
	35b. If variable compensation changed over time, how did it vary?
35c. Outline the variable compensation scheme that was used at the end of the grant. If it different in implementation areas, please outline how it differed:	
35d. On average, how much variable compensation did CHWs earn per month in the project?	________ USD
35e. Please provide the **minimum** variable compensation that was provided during the grant:	________ USD
35f. Please provide the **maximum** variable compensation that was provided during the grant:	________ USD

Compensation- non-monetary compensation

36. Please select all of the non-monetary benefits provided as compensation to the CHWs in your project ***(check all that apply):***	☐ Food<br>☐ Supplements<br>☐ Stationary<br>☐ Clothing<br>☐ Mode of transport (motorbike, car, bicycle)<br>☐ Right to retain project technology such as tablets or phones<br>☐ Priority access to TB/HIV or other disease testing<br>☐ Health insurance<br>☐ Life insurance<br>☐ Other _____________

37. Please list any other non-monetary compensation that was provided by the project.	
38. What was the total cost of non-monetary compensation in your grant? (Total budget line for non-monetary compensation)	___________ USD<br>☐ Unknown

Supervision of CHWs

39. What was the supervisory structure in your project? (For example, 2 Directors-> 4Project Managers-> 16Field Coordinator -> 140 CHWs)	
40. Could performance issues be addressed by the direct supervisor or would it be elevated to a higher level of management?	☐ Performance issues addressed by direct supervisor<br>☐ Performance managed addressed by upper management
41. What was the gender breakdown of the CHWs' supervisors employed in the project?	______% Male<br>______% Female<br>______% Other<br><br>Please specify how you define other:
42. On average, how many CHWs would one supervisor directly oversee?	
43. Did you use paper or electronic monitoring tools to track the activities and performance of the CHWs?	☐ Paper-based tools<br>☐ Electronic tools<br>☐ Both
44. Specifically, which tools did you use to track the activities and performance of the CHWs? (Check all that apply)	☐ Monitoring forms<br>☐ Screening data<br>☐ GPS data<br>☐ Timesheets<br>☐ Financial data<br>☐ Verification with external stakeholders (Such as clinic staff)<br>☐ Lab registers<br>☐ Routine surveillance data<br>☐ Customer Relationship Management Software (CRM)<br>☐ Other______________________________
45. On average, how often would supervisors review the work of a CHW per quarter?	___________ times per quarter
46. On average, how often would supervisors provide direct verbal feedback to the CHWs they supervised?	___________ times per quarter

Refresher training(s)

47. How often did you conduct refresher training during the duration of the grant?	___________ times during the grant
48. On average, for how many hours was a refresher training for a CHW?	______ hours
49. Did all CHWs receive refresher training during the grant duration?	☐Yes ☐No ☐Unknown
	49a. If no, who received refresher training?
	☐ Formal

50. Were refresher trainings formal or informal?	☐ Informal<br>☐ No refresher training was conducted
	50a. If formal, what were the formal modes of refresher training? (check all that apply)<br>☐ Face-to-face with an expert trainer in a classroom setting<br>☐ Face-to-face with an expert trainer in a community setting<br>☐ Peer-to-peer training in a classroom setting<br>☐ Peer-to-peer training in a community setting<br>☐ e-learning<br>☐ Hands on practice of skills in a classroom setting<br>☐ Hands on practice of skills in a community setting<br>☐ Other ______________
51. Were the CHWs compensated for participating in the refresher training?	☐Yes ☐No ☐Unknown
	51a. If yes, check the types of compensation:<br>☐ Cash per diem<br>☐ Food during training<br>☐ Other ______________
	51b. What was the average cost per participant of participating in the refresher-service training? _________

CHW upward mobility

52. Were any CHWs promoted to a higher role within your organization during or after implementation of the TB REACH grant?	☐Yes ☐No ☐Unknown
	52a. If yes, how many were promoted?
	52b. To what roles were they promoted?

Sustainability of the CHW model

53. Did the CHWs working on the TB REACH project keep their jobs at the close of the project?	☐Yes, all CHWs kept their jobs<br>☐Yes, a subset of CHWs kept their jobs<br>☐No ***If no, please skip to the next section**<br>☐Unknown ***If unknown, please skip to the next section**
53a. Did the CHWs continue working with the same roles and responsibilities as during the TB REACH grant?	☐Yes, they continued with the same job<br>☐No, they continued but with different roles and responsibilities<br>☐Unknown
53b Did the management structure remain the same as during the TB REACH grant?	☐Yes ☐No ☐Unknown
53c Did the compensation package remain the same as during the TB REACH grant?	☐Yes ☐No ☐Unknown
53d. Which funding agency or donor continued to support the work of the CHWs at the close of the TB REACH project?	

Additional information

54. Is there anything else you would like to add?	

Thank you for your time!
